# Metabolic Syndrome in Patients with Polycystic Ovary Syndrome in Iran

**DOI:** 10.22074/ijfs.2015.4607

**Published:** 2015-12-23

**Authors:** Ziba Zahiri, Seyedeh Hajar Sharami, Forozan Milani, Fereshteh Mohammadi, Ehsan Kazemnejad, Hannan Ebrahimi, Seyedeh Fatemeh Dalil Heirati

**Affiliations:** 1 Reproductive Health Research Center, Obstetrics and Gynecology Department, Alzahra Hospital, Guilan University of Medical Sciences, Rasht, Iran; 2 Guilan University of Medical Sciences, Rasht, Iran

**Keywords:** Polycystic Ovary Syndrome, Metabolic Syndrome, Prevalence

## Abstract

**Background:**

The prevalence of metabolic syndrome (MetS) in polycystic ovary syndrome (PCOS) has been studied in different populations, but their results were so controversial regarding Iranian women. These controversial data indicated the need for more investigation of MetS characteristics in PCOS patients in our population. So this study aimed to evaluate the clinical and laboratory characteristics and metabolic features of patients with PCOS in Rasht.

**Materials and Methods:**

This prospective cross sectional study was conducted on 215 PCOS women who lived in Rasht, north of Iran, from March 2010 to July 2012. The participants were then divided into two groups of women with MetS (n=62) and women without MetS (n=153). The diagnosis of PCOS and MetS were based on the Rotterdam 2003 criteria and the Adult Treatment Panel III (ATP III) criteria, respectively. Demographic characteristics, fertility characteristics, family history and laboratory findings were assessed.

**Results:**

The prevalence of MetS in women with PCOS was 28.8%. In PCOS women of both groups, the waist circumference (WC) exceeded 88cm in 72.6%, hypertension [systolic blood pressure (SBP) and/or diastolic blood pressure (DBP) ≥130/85mm Hg] was prevalent in 9.3%, fasting blood sugar (FBS) level was ≥110 mg/dl in 6%, triglycerides (Tg) level were ≥150 mg/dl in 47%, and high-density lipoprotein (HDL) level was <50 mg/dl in 86%. The values of WC, SBP, DBP, body mass index (BMI), ovarian size, Tg, cholesterol, FBS, 2-hour blood sugar, aspartate aminotransferase (AST), and alanine aminotransferase (ALT) were significantly greater in PCOS women with MetS than women without MetS. Also HDL and luteinizing hormone (LH) levels in women with MetS were significantly lower than women without MetS.

**Conclusion:**

Prevalence of MetS in PCOS women was 28.8%, indicating that this value is higher than other studies conducted on PCOS women in Iran and other studies conducted on general population in Iran. PCOS women are considered as a high-risk population for MetS. The special strategies are required to prevent MetS and its associated complications in PCOS women.

## Introduction

Polycystic ovary syndrome (PCOS) is one of the most common gynecological endocrinopathy among reproductive-aged women (1, 2). The prevalence of PCOS have been reported from 2.2 to 26% in different studies conducted in various countries, depending on sampling method , the criteria used for its definition and the method used to define each criterion (1-3). The clinical and biochemical features of PCOS may vary according to race, ethnicity and the diagnostic criteria used (4). In a study by Tehrani et al. (3), the prevalence of PCOS in a community sample of Iranian population was 7.1% using the National Institute of Health (NIH) definition, 11.7% by the Androgen Excess Society (AES) criteria and 14.6% using the Rotterdam consensus definition. The classic form of PCOS is characterized by chronic anovulation (oligomenorrhea or amenorrhea), hyperandrogenism, infertility, hirsutism, obesity and enlarged bilateral ovaries with cysts (5-7). The pathogenesis of PCOS is not fully understood, although genetic, metabolic and neuroendocrine interactions as well as environmental factors were discussed elsewhere (8-11). Alterations in several metabolic pathways such as steroid hormone regulation and insulin signaling pathway abnormalities were discussed in the pathophysiology of PCOS (12-14). More than 50 percent of women with PCOS are obese or overweight that may predispose them to metabolic disorders (15).

The metabolic syndrome (MetS) is one type of endocrine disturbance that consists of insulin resistance, dyslipidemia, obesity, central adiposity, and hypertension that has been shown to be associated with a two-fold increased risk of cardiovascular disease and a five-fold increased risk of type 2 diabetes (16). The prevalence of MetS in PCOS has been studied in different populations (16-19). In a study conducted in the USA showed that the prevalence of MetS in PCOS women was approximately 43 to 46%, while for aged-matched women in the general population, it was nearly 2-fold higher (16). However, the findings of several studies conducted in Iran regarding the prevalence of MetS in PCOS women were controversial (18-20). Hosseinpanah et al. (18) showed that MetS was less frequent in patients with PCOS. However, in other studies, MetS was noted in younger PCOS patients in comparison with older PCOS women (19, 20). These controversial data indicate the need for more investigation of MetS characteristics in PCOS patients in our population, as it may help in planning screening strategies to prevent long-term effects (17). Therefore, this study aimed to evaluate the clinical and laboratory characteristics and metabolic features of patients with PCOS in Rasht, North of Iran.

## Materials and Methods

This prospective cross sectional study was conducted on 215 PCOS women aged 15-35 years who were referred to private and public gynecological endocrinology clinics, Rasht, north of Iran, from March 2010 to July 2012. The exclusion criteria were as follows: lactating and pregnant women, previous history of ovarian surgery, use of steroid hormone drugs such as oral contraceptive pill and progesterone for past 6 months, use of dyslipidemia drugs for last 3 months, and use of any medications known to affect glucose metabolism or BP. Furthermore women with following conditions were excluded: hypothyroidism, hyperprolactinemia, congenital adrenal hyperplasia, androgen- producing tumor, and Cushing’s syndrome that were diagnosed by physical examination and laboratory testing using serum levels of thyroid stimulating hormone (TSH), prolactin (PRL), and 17a-hydroxyprogesterone (17a-OHP). All patients provided a written informed consent before entering the study. The participants were then divided into two groups of women with MetS (n=62) and women without MetS (n=153). The study protocol was approved by the Ethics Committee of Guilan University of medical sciences, Rasht, Iran.

The diagnosis of PCOS was based on the Rotterdam 2003 criteria, in which any two of the following three conditions need to be fulfilled for the inclusion: i. Oligo- and/or anovulation (i.e. less than 9 menstrual periods in a year or menstrual cycles more than 35 days in length), ii. Clinical hyperandrogenism (i.e. acne or hirsutism; modified Ferriman-Gallwey scores ≥8) or biochemical hyperandrogenism [i.e. free testosterone (FT) ≥7.0 pg/ml], and iii. Ultrasonographic findings of polycystic ovarian morphology (presence of ≥12 follicles in each ovary measuring 2-9 mm in diameter). Based on these criteria, four phenotypes were formed as follows: type 1 including irregular menstruation+PCO using ultrasonographic examination+hyperandrogenism (IM+PCO+HA), type 2 including irregular menstruation+PCO using ultrasonographic examination (IM+PCO), type 3 including irregular menstruation+hyperandrogenism (IM+HA) and type 4 including PCO using ultrasonographic examination+hyperandrogenism (PCO+HA).

MetS was defined according to the Adult Treatment Panel III (ATP III) criteria as the co-occurrence of three or more of the following risk factors: i. Central obesity with waist circumference (WC) ≥88 cm in women, ii. Elevated systolic blood pressure (SBP) and/or diastolic blood pressure (DBP) of ≥130/85 mmHg, iii. Impaired level of fasting blood sugar (FBS) ≥110 mg/dL, iv. Elevated level of fasting serum triglycerides (Tg) ≥150 mg/dL and v. Fasting high-density lipoprotein (HDL) level <50 mg/dL.

Anthropometric measurements included height in centimeters, weight in kilograms, and hip and waist circumference in centimeters according to World Health Organization (WHO) categories. Body mass index (BMI) was calculated as weight in kilograms divided by the square of height in meters (kg/m^2^). Underweight was defined as less than 18.5 (normal range between 18.5 and 24.9), overweight between 25.0 and 29.9 and obese as 30.0 or higher. Sitting blood pressure was measured after a 5-minute rest using a standard sphygmomanometer.

After 12 hours fasting during days 3-5 of menstrual cycle, 10 cc of blood sample was obtained. Follicle-stimulating hormone (FSH) and luteinizing hormone (LH) levels were measured using Access Immunoassay System (Beckman Coulter, Fullerton, California, USA); FT, dehydroepiandrosterone sulfate (DHEAS), and 17a-OHP levels using radio-immunometric assay (RIA) kit (Siemens, USA); as well as TSH level using a chemiluminescence immunometric assay (Immulite 2000 Analyzer, CPC, USA). Also fasting blood sample was used to measure FBS level using Hitachi 7600 analyzer, Hitachi, Japan, while the levels of cholesterol (Chol), Tg, aspartate aminotransferase (AST), alanine aminotransferase (ALT), HDL, and low-density lipoprotein (LDL) were measured using enzymatic calorimetric method (Hitachi 7600). In addition a 75-gram oral glucose tolerance test was measured.

Demographic variables including age, education, occupation, and inhabitant area; reproductive characteristics including parity, history of infertility, type of menstrual irregularity such as oligomenorrhea, amenorrhea, and menometrorrhagia; and family history of diabetes mellitus were collected.

### Statistical analysis

All data were analyzed using the Statistical Package for the Social Sciences (SPSS, SPSS Inc., USA) software version 16. Continues data were shown as mean ± standard deviation (SD). Kolmogorov-Smirnov test was used to assess the normality of continuous variable. Normal distribution of quantitative variables was analyzed by two-tailed independent t test and non-normally distributed variables by Mann-Whitney U test. Categorical data were shown as number (percentage). Fisher’s exact tests and chi-square test were used to compare the groups. Statistical significance was considered as P<0.05.

## Results

Prevalence of MetS in women with PCOS was 28.8%. In PCOS women of both groups (15-35 years of age), the mean age was 25.63 ± 5.17. In this study, 72 patients were single and 123 were married, but in the analysis of reproductive characteristic, investigators did not assess single patients.

Most of women (83.8%) lived in urban area. Majority (79.7%) of women were housewife and some (38.4%) of women had a university degree. Mean infertility duration was 36.50 ± 41.26. About 54.9, 10.6, and 39.3% of married patients showed a history for infertility, abortion, and diabetes in their families, respectively. Twenty two percent of married women were multiparous. Oligomenorrhea was reported in 85.1% of women. Except family history of diabetes (P=0.043), there were no significant differences regarding demographic and fertility characteristics between PCOS women with MetS and without MetS (Table 1).

The findings of both groups showed that the WC exceeded 88 cm in 72.6%, hypertension (SBP/ DBP ≥130/85 mm Hg) in 9.3%, FBS level was 110 mg/dl or greater in 6%, Tg level was 150 mg/dl or greater in 47%, and HDL level was less than 50 mg/dl in 86%. Individual components of the MetS in two groups of PCOS women (with MetS and without MetS) are shown in table 2.

**Table 1:** Comparison of demographic and fertility characteristics between polycystic ovary syndrome women with and without metabolic syndrome (MetS)


Variables	Total (n=215)	With MetS (n=62)	Without MetS (n=153)	P value

Age (Y)	25.63 ± 5.17	26.81 ± 6.07	25.15 ± 4.69	0.057
Inhabitation areaRural	34 (16.2)	9 (14.5)	25 (16.9)	0.838
Urban	176 (83.8)	53 (85.5)	123 (83.1)	
JobEmployed	32 (20.3)	11 (24.4)	21 (18.6)	0.511
Housewife	126 (79.7)	34 (75.6)	92 (81.4)	
Educational levelUnder diploma	42 (26.4)	12 (27.3)	30 (26.1)	0.950
Diploma	56 (35.2)	16 (36.4)	40 (34.8)	
University	61 (38.4)	16 (36.4)	45 (39.1)	
Duration of infertility	36.50 ± 41.26	30.39 ± 24.04	39.42 ± 47.21	0.705
History of infertility	95 (54.9)	30 (58.8)	65 (68.4)	0.615
History of abortion	13 (10.6)	5 (13.5)	8(9.0)	0.523
Family history of diabetes	83 (39.3)	31(50.8)	52 (34.7)	0.043
History of parityNulliparous	96 (78)	24 (75)	59 (77.6)	0.760
Multiparous	27 (22)	8 (25)	17 (22.4)	
Oligomenorrhea	183 (87.6)	52 (89.7)	131(86.8)	0.647
Amenorrhea	20 (9.3)	6 (10.3)	14 (9.3)	0.797
Polymenorrehae	6 (2.8)	0	6 (4)	0.190
Normal	6 (2.8)	4(6.5)	2 (1.3)	0.059


Data are presented as mean ± SD or numbers (%).

**Table 2:** Comparison of prevalence of individual components of the metabolic syndrome (MetS) between polycystic ovary syndrome women with and without MetS


Components of the MetS	Total (n=215)	With MetS (n=62)	Without MetS (n=153)

WC ≥88 cm	156 (72.6%)	59 (95.2)	97 (63.4)
Hypertension (SBP ≥130 mm Hg or DBP ≥85 mm Hg)	20 (9.3)	17 (27.4)	3 (2.0)
FBS ≥110 mg/dl	13 (6.0)	12 (19.4)	1 (7.7)
Tg ≥150 mg/dl	101 (47.0)	55 (88.7)	46 (30.1)
HDL ˂50 mg/dl	185 (86.0)	61 (98.4)	124 (81.0)


Data are presented as numbers (%). WC; Waist circumference, SBP; Systolic blood pressure, DBP; Diastolic blood pressure, Tg; Triglycerides, FBS; Fasting blood sugar and HDL; high-density lipoprotein.

In all patients, mean values of WC, SBP, DBP, and BMI were 87.37 ± 12.38, 110.32 ± 14.72, 69.90 ± 9.64, and 28.98 ± 11.19, respectively. Mean levels of Tg, FBS, and HDL were 152.39 ± 74.29, 93.02 ± 17.79, and 41.33 ± 8.64, respectively. Mean values of WC, SBP, DBP, and BMI in patients with MetS were significantly higher than women without MetS (Table 3).

Some laboratory findings including Tg, Chol, FBS, 2-hBS, AST, and ALT were significantly greater in PCOS women with MetS than women without MetS. Also HDL and LH in women with MetS were significantly lower than women without MetS. In other laboratory findings, such as serum levels of LDL, PRL, DHEAS, T, FSH, FSH/ LH, 17OHP, and TSH, differences between two groups were not significant (Table 4).

Prevalence of MetS in PCO+HA phenotype was highest, but this difference was not statistically significant among four PCOS subtypes (Table 5).

**Table 3:** Comparison of anthropometric and BP measurements between polycystic ovary syndrome women with and without metabolic syndrome (MetS)


Components of the MetS	Total (n=215)	With MetS (n=62)	Without MetS (n=153)	P value

WC (cm)	87.37 ± 12.38	97.24 ± 11.14	83.37 ± 10.51	0.0001
BMI (kg/m^2^)	28.98 ± 11.19	32.92 ± 10.80	27.37 ± 10.94	0.001
BMI ≥30	77 (36)	42 (67.7)	35 (23)	0.0001
SBP (mmHg)	110.32 ± 14.72	116.62 ± 15.64	107.77 ± 13.56	0.0001
DBP (mmHg)	69.90 ± 9.64	72.42 ± 10.93	68.88 ± 8.91	0.015


Data are presented as mean ± SD. WC; Waist circumference, BMI; Body mass index, SBP; Systolic blood pressure and DBP; Diastolic blood pressure.

**Table 4:** Comparison of laboratory findings between polycystic ovary syndrome women with and without metabolic syndrome (MetS)


Variables	Total (n=215)	With MetS (n=62)	Without MetS (n=137)	P value

Tg (mg/dl)	152.39 ± 74.29	202.76 ± 90.69	132.0 ± 54.93	0.0001
Chol (mg/dl)	180.37 ± 37.08	195.18 ± 36.27	174.33 ± 35.80	0.0001
FBS (mg/dl)	93.02 ± 17.79	99.97 ± 29.37	90.20 ± 8.46	0.012
LDL (mg/dl)	111.72 ± 28.00	117.42 ± 30.59	109.40 ± 26.63	0.057
HDL (mg/dl)	41.33 ± 8.64	37.13 ± 6.31	43.03 ± 8.88	0.0001
2-h BS (mmol/l)	112.90 ± 37.79	134.28 ± 54.21	104.50 ± 24.53	0.001
AST (mg/dl)	22.47 ± 10.57	25.76 ± 10.23	21.12 ± 10.45	0.003
ALT (mg/dl)	20.42 ± 12.94	23.53 ± 9.94	19.15 ± 13.81	0.024
PRL (ng/mL)	19.33 ± 11.44	20.47 ± 14.52	18.85 ± 9.94	0.349
DHEAS (mg/dl)	237.45 ± 136.88	228.04 ±145.99	241.20 ± 133.38	0.527
FT (nmol/l)	1.72 ± 1.59	1.89 ± 2.42	1.65 ± 1.10	0.467
FSH (mg/dl)	6.17 ± 1.77	6.01 ± 2.08	6.22 ± 1.65	0.494
LH (mg/dl)	7.89 ± 4.12	6.79 ± 1.64	8.29 ± 4.66	0.002
LH/FSH	1.34 ± 0.66	1.26 ± 0.53	1.38 ± 0.70	0.251
17OHP	1.91 ± 14.31	0.85 ± 0.57	2.34 ± 16.91	0.494
TSH	3.43 ± 7.75	4.95 ± 13.67	2.82 ± 2.91	0.228


Data are presented as mean ± SD. Tg; Triglycerides, Chol; Cholesterol, FBS; Fasting blood sugar, LDL; Low-density lipoprotein, HDL; Highdensity lipoprotein, 2-h BS; 2-hour blood suger, AST; Aspartate aminotransferase, ALT; Alanine aminotransferase, PRL; Prolactin, DHEAS; Dehydroepiandrosterone sulfate, FT; Free testosterone, FSH; Follicle-stimulating hormone, LH; Luteinizing hormone, 17OHP; 17hydroxyprogesterone and TSH; Thyroid stimulating hormone.

**Table 5:** Prevalence of metabolic syndrome (MetS) in four polycystic ovary syndrome (PCOS) subtypes


PCOS subtype	With MetS (n=62)	Without MetS (n=137)	P value

IM+PCO+HA	38 (32.8)	78(67.2)	0.138
IM+PCO	9 (33.3)	18 (66.7)	
IM+HA	10 (17.2)	48 (82.8)	
PCO+HA	5 (38.5)	8 (61.5)	


IM; Irregular menstruation, PCO; Polycystic ovary and HA; Hyperandrogenism.

## Discussion

PCOS is one of the most important endocrine diseases in women. Many PCOS patients with several metabolic abnormalities are at increased risk of MetS. The prevalence of MetS differs in various populations which is mainly due to definition of PCOS or MetS, sampling methods, selecting controls, as well as age, race and weight of participants. Insulin resistance (IR) plays a crucial role in the pathophysiology of MetS. On the other hand, IR is well recognized to play a major role in the etiology of PCOS (21-23).

As expressed in previous reports, the prevalence of MetS in patients with PCOS is higher than general population (16, 18, 24), but its prevalence is not the same in different ethnic groups. Increased waist circumferences, elevated Tg level and reduced HDL level, known as important components of MetS, are associated with genetic factors and lifestyle characteristics (16, 24). The finding of an increased risk of MetS in PCOS women has raised further interest in identifying the predictors for MetS in these women (25). This study examined the prevalence and related factors of MetS in PCOS patients living in north of Iran.

In this study, the prevalence of MetS in PCOS women was 28.8%. This finding is higher than other studies in Iran (4, 18, 24), but lower than studies in United States (26, 27). Mehrabian et al. (4) showed that the prevalence of MetS were 24.9% among Iranian women diagnosed with different phenotypic subgroups of PCOS, based on the Rotterdam criteria. Moini et al. (24) who conducted a study in Tehran, Iran, reported that prevalence of MetS in PCOS women was 22.7%. In another large-scale population-based study in Tehran, the prevalence of MetS in PCOS subjects was 18.5% (18). This different prevalence may be related to population characteristics, diagnostic criteria and sample size.

This study showed that values of WC, SBP, DBP, BMI, obesity, and ovarian size were significantly greater in patients with MetS than patients without MetS. In a report by Mandrelle et al. (17), age and central obesity (waist-hip ratio/waist circumference) were considered as better predictors of MetS in PCOS women as compared to other parameters including BMI in this group of women.

This study showed that the values of biochemical parameters such as Tg, Chol, FBS, 2h BS, AST, and ALT were significantly greater in patients with MetS than without MetS. Also HDL and LH levels in women with MetS were significantly lower than women without MetS. Soares et al. (16) reported that the occurrence of low HDL was the most frequent individual component of MetS among Brazilian women with PCOS, followed by increased serum Tg.

For study limitation, our findings may be influenced by the criteria, by which PCOS and MetS were diagnosed. We used different equipment and different assessors during our study period that may affect our assessment reliability. Also this study was a cross sectional study with small sample size. So it is suggested to do more cohort or case control studies with greater sample size in future.

## Conclusion

Based on our findings, prevalence of MetS in PCOS women was 28.8% that was higher than related values of other studies conducted on both PCOS women and general population in Iran. Among individual components of MetS, WC>88 cm, HDL>50, and Tg ≥150 were prevalent in more than 88% of PCOS women. PCOS women are high risk population for MetS. The special strategies is required for prevention of MetS and its related complications in PCOS women.
